# Physician Decision-Making Patterns and Family Presence: Cross-Sectional Online Survey Study in Japan

**DOI:** 10.2196/12781

**Published:** 2019-09-06

**Authors:** Kenji Tsuda, Asaka Higuchi, Emi Yokoyama, Kazuhiro Kosugi, Tsunehiko Komatsu, Masahiro Kami, Tetsuya Tanimoto

**Affiliations:** 1 Medical Governance Research Institute Minatoku Japan; 2 Graduate School of Education, Seisa University Yokohama Japan; 3 National Cancer Center Hospital East Kashiwa Japan; 4 Jyoban Hospital of Tokiwa Foundation Iwaki Japan; 5 Navitas Clinic Tachikawa Japan

**Keywords:** physician decision making, family, cross-sectional study, cardiopulmonary resuscitation, artificial ventilation

## Abstract

**Background:**

Due to a low birth rate and an aging population, Japan faces an increase in the number of elderly people without children living in single households. These elderly without a spouse and/or children encounter a lack of caregivers because most sources of care for the elderly in Japan are not provided by private agencies but by family members. However, family caregivers not only help with daily living but are also key participants in treatment decision making. The effect of family absence on treatment decision making has not been elucidated, although more elderly people will not have family members to make surrogate decisions on their behalf.

**Objective:**

The aim is to understand the influence of family absence on treatment decision making by physicians through a cross-sectional online survey with three hypothetical vignettes of patients.

**Methods:**

We conducted a cross-sectional online survey among Japanese physicians using three hypothetical vignettes. The first vignette was about a 65-year-old man with alcoholic liver cirrhosis and the second was about a 78-year-old woman with dementia, both of whom developed pneumonia with consciousness disturbance. The third vignette was about a 70-year-old woman with necrosis of her lower limb. Participants were randomly assigned to either of the two versions of the questionnaires—with family or without family—but methods were identical otherwise. Participants chose yes or no responses to questions about whether they would perform the presented medical procedures.

**Results:**

Among 1112 physicians, 454 (40.8%) completed the survey; there were no significant differences in the baseline characteristics between groups. Significantly fewer physicians had a willingness to perform dialysis (odds ratio [OR] 0.55, 95% CI 0.34-0.80; *P*=.002) and artificial ventilation (OR 0.51, 95% CI 0.35-0.75; *P*<.001) for a patient from vignette 1 without family. In vignette 2, fewer physicians were willing to perform artificial ventilation (OR 0.59, 95% CI 0.39-0.90; *P*=.02). In vignette 3, significantly fewer physicians showed willingness to perform wound treatment (OR 0.51, 95% CI 0.31-0.84; *P*=.007), surgery (OR 0.35, 95% CI 0.22-0.57; *P*<.001), blood transfusion (OR 0.45, 95% CI 0.31-0.66; *P*<.001), vasopressor (OR 0.49, 95% CI 0.34-0.72; *P*<.001), dialysis (OR 0.38, 95% CI 0.24-0.59; *P*<.001), artificial ventilation (OR 0.25, 95% CI 0.15-0.40; *P*<.001), and chest compression (OR 0.29, 95% CI 0.18-0.47; *P*<.001) for a patient without family.

**Conclusions:**

Elderly patients may have treatments withheld because of the absence of family, highlighting the potential importance of advance care planning in the era of an aging society with a declining birth rate.

## Introduction

With the progress of a low birth rate and an aging population, Japan faces an increase of elderly people without children living in single households. In 2016, 4% of people aged 60 years or older did not have children and were living alone [1]. These elderly without spouses and/or children result in a lack of caregivers because most care for the elderly in Japan is not provided by private agencies but by family members. Sixty percent of care is provided by family members living together, half of which is done by the spouse and the other half by children or spouses of children [2].

Family caregivers are not only helpers of daily living but are key participants in the treatment decision making [3]. More than one-quarter of hospitalized patients lack the capacity to make decisions about their care because of the illness itself or chronic cognitive disorders such as dementia. As such, family members often play the role of surrogates to make decisions on behalf of the patients [4]. For intensive care unit physicians, family directives were the fifth most important factor in withdrawal of life support and valued more than age and premorbid physical function of patients [5]. However, 16% of patients in intensive care units and 3% of nursing home residents had no designated surrogate and no identifiable family members to speak on their behalf [4,6,7]. In such cases, decisions to limit life support are generally made by physicians [6,8].

The effect of family absence on physician decision making has not been elucidated. Therefore, we conducted a cross-sectional online survey with three hypothetical vignettes of patients to estimate the influence of family absence on treatment decision making by Japanese physicians.

## Methods

On February 2018, we distributed an online survey via email to all Japanese physicians registered to the mailing list offering job information provided by Mediwel Co, Ltd. In Japan, approximately 60% of physicians take on a part-time job in addition to their full-time job, and many physicians register to this mailing list as a source of information. The coverage of the database is not open to the public, but it is rated as one of the largest platforms for job-seeking in Japan. This is the first survey using this mailing list for research. Participation in our survey was voluntary without any incentives.

For the baseline characteristics, physicians provided their age, sex, years since graduation, type of institution that they worked in, number of times they have explained a patient’s condition to his or her family per year, and specialty. No other personal information was collected.

Using the online survey, anonymity was secured, and physicians could answer honestly without fear of reputational risk.

Participants were informed that the aim of the study was to investigate physicians’ decision making. To avoid biases, we did not mention the survey purpose was to investigate the effect of family absence. Participants were randomly assigned to either of two versions of the vignettes: without family (pattern A) or with family (pattern B). Each pattern included three vignettes; these were identical except for the description of family presence or absence. Allocation was randomized through the A/B testing function of the mail delivery system, which is often used in internet marketing to compare two versions of a webpage. The deadline for answers was one week later.

Details of the vignettes are shown in Multimedia Appendices 1 and 2. Briefly, the first vignette described a 65-year-old man with alcoholic liver cirrhosis, and the second described a 78-year-old woman with advanced dementia. Both of these patients developed pneumonia with impairment of consciousness and were taken to a hospital by an ambulance. In vignettes 1 and 2, patients did not have an advanced directive, and their preferences for treatment were unknown. The third vignette described a 70-year-old woman with necrosis of her lower limb, which needed amputation to save her life. The patient did not want amputation nor cardiopulmonary resuscitation. However, in pattern B, the woman’s family wanted all possible treatments even though the patient refused to receive the procedures. To each vignette, respondents chose yes or no answers to questions for approximately 10 medical procedures about whether they would perform procedures necessary for the patient.

The primary endpoint was not set because there were no similar previous studies. This was intended as an exploratory survey, but we targeted for at least 100 participants in each pattern. All acquired data were analyzed, and no kinds of sampling were performed. Statistical analyses were performed using R version 3.3.3 (R Foundation for Statistical Computing, Vienna, Austria). Between patterns A and B, differences in age and years since graduation were tested by Student *t* tests; other baseline characteristics were tested by chi-square tests. Physicians’ choices were compared between patterns A and B for each procedure by chi-square tests and Fisher exact tests. Subanalyses investigating differences in sex, experience, and type of institution were performed for procedures that showed significant differences between patterns A and B. Experienced physicians were defined as aged 41 years or older. Univariate analysis was performed, and factors related to withholding of artificial ventilation were identified. All variables with a *P* value <.10 in the initial univariate analysis were considered a potential influencing factor in the multivariate logistic regression model. A *P* value of <.05 was considered statistically significant. This study was approved by the institutional review board of the Medical Governance Research Institute, Tokyo, Japan.

## Results

**Figure 1:**
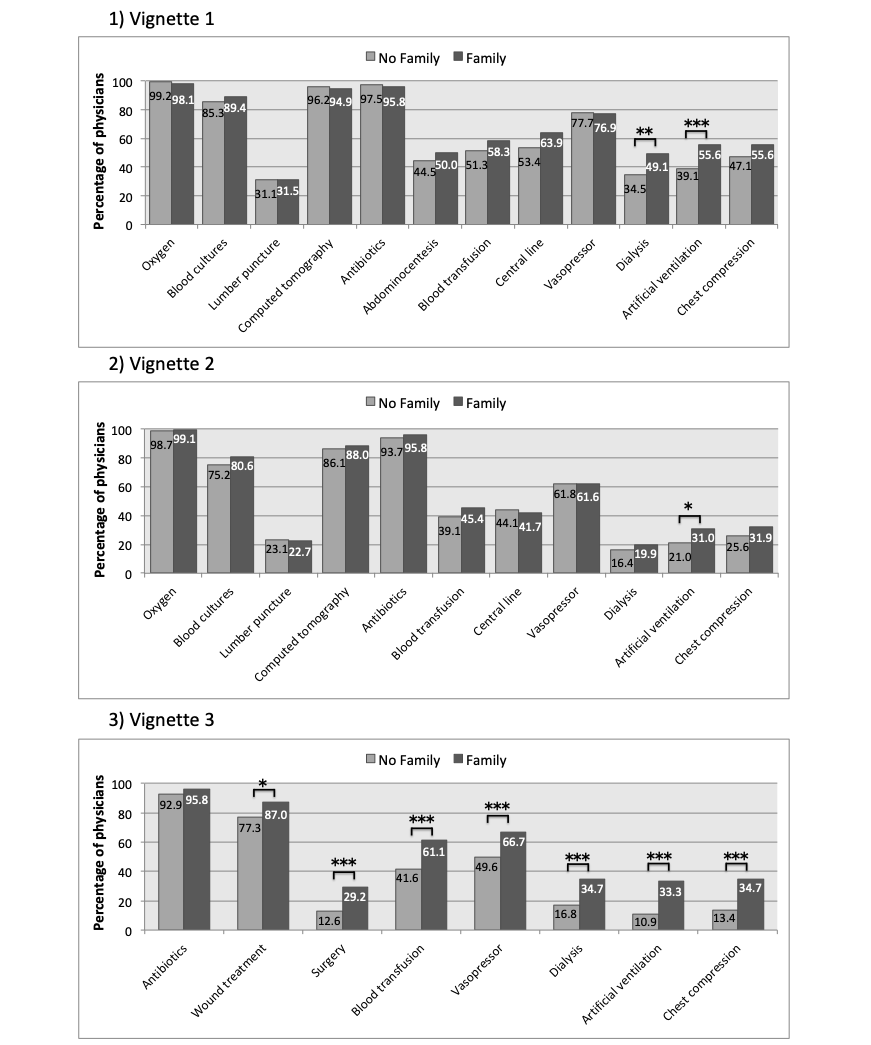
Differences in physicians’ decision-making patterns between the presence and absence of family for critically ill patients. *P<.05, **P<.01, ***P<.001.

Among 1112 physicians who opened the link attached to the invitation email, 454 (40.8%) physicians completed the survey. There were no significant differences in the baseline characteristics between groups (Table 1). In vignette 1, for a patient without family compared with a patient with family, significantly fewer physicians were willing to perform dialysis (odds ratio [OR] 0.55, 95% CI 0.34-0.80; *P*=.002) and artificial ventilation (OR 0.51, 95% CI 0.35-0.75; *P*<.001). In vignette 2, fewer physicians were willing to perform artificial ventilation (OR 0.59, 95% CI 0.39-0.90; *P*=.02; Figure 1). Similarly, in vignette 3, significantly fewer physicians were willing to perform wound treatment (OR 0.51, 95% CI 0.31-0.84; *P*=.007), surgery (OR 0.35, 95% CI 0.22-0.57; *P*<.001), blood transfusion (OR 0.45, 95% CI 0.31-0.66; *P*<.001), vasopressor (OR 0.49, 95% CI 0.34-0.72; *P*<.001), dialysis (OR 0.38, 95% CI 0.24-0.59; *P*<.001), artificial ventilation (OR 0.25, 95% CI 0.15-0.40; *P*<.001), and chest compression (OR 0.29, 95% CI 0.18-0.47; *P*<.001) for a patient without family.

As shown in Table 2, male physicians significantly withheld all 10 procedures in patients without family. For female physicians, the odds ratios of all 10 procedures were less than 1, but only a few procedures were statistically significant. This was probably because there were fewer female participants compared with male participants in this study (n=84 and n=370, respectively). Table 3 shows that experienced physicians significantly withheld all 10 procedures in patients without family. For nonexperienced physicians, odds ratios of all 10 procedures were less than 1, but only a few procedures were statistically significant. As shown in Table 4, physicians working at a non-acute care hospital significantly withheld all 10 procedures in patients without family. In physicians working at an acute care hospital, the odds ratios of all 10 procedures were less than 1, but only a few procedures were statistically significant.

As shown in Table 5, in univariate analysis, years since graduation and pattern were found to be associated with withholding artificial ventilation in vignette 1. Institution and pattern were significant in vignette 2, and only pattern was significant in vignette 3. In a multivariate analysis, pattern was the only factor that was associated with withholding artificial ventilation through all vignettes.

**Table 1:** Baseline characteristics of study participants (N=454).

Variable		No family (n=238)	Family (n=216)	*P* value
Male, n (%)		194 (81.5)	176 (81.5)	>.99
Age (years), mean (SD)		45.7 (10.2)	45.1 (9.5)	.54
Years since graduation, mean (SD)		19.8 (9.9)	19.1 (9.1)	.42
**Institution, n (%)**				**.20**
	Acute care hospital	132 (55.5)	132 (61.1)	
	Clinic	51 (21.4)	48 (22.2)	
	Chronic care hospital	32 (13.4)	26 (12.0)	
	Other	23 (9.7)	10 (4.6)	
**Number of times they have explained patient’s condition to his or her family per year, n (%)**				**.75**
	0	13 (5.5)	11 (5.1)	
	1-6	32 (13.4)	37 (17.1)	
	7-12	22 (9.2)	19 (8.8)	
	13 or more	171 (71.8)	149 (69.0)	
**Specialty, n (%)**				**.11**
	Internal medicine	95 (39.9)	88 (40.7)	
	Surgery	27 (11.3)	14 (6.5)	
	Orthopedics	24 (10.1)	11 (5.1)	
	Psychiatry	18 (7.6)	14 (6.5)	
	Others	74 (31.1)	89 (41.2)	

**Table 2:** Odds ratios of performing procedures in pattern A (without family present) versus pattern B (with family present) by sex (N=454).

Vignette and procedure		Male (n=370)		Female (n=84)	
		Odds ratio (95% CI)	*P* value	Odds ratio (95% CI)	*P* value
**Vignette 1**					
	Dialysis	0.52 (0.34-0.78)	.002	0.69 (0.29-1.64)	.51
	Artificial ventilation	0.48 (0.32-0.73)	<.001	0.67 (0.28-1.58)	.39
**Vignette 2**					
	Artificial ventilation	0.57 (0.35-0.91)	.02	0.70 (0.28-1.76)	.49
**Vignette 3**					
	Wound treatment	0.48 (0.28-0.84)	.01	0.64 (0.19-2.16)	.55
	Surgery	0.36 (0.21-0.62)	<.001	0.30 (0.09-0.95)	.06
	Blood transfusion	0.48 (0.31-0.72)	<.001	0.36 (0.14-0.88)	.03
	Vasopressor	0.52 (0.34-0.79)	.002	0.37 (0.14-0.92)	.04
	Dialysis	0.34 (0.2-0.55)	<.001	0.61 (0.23-1.61)	.34
	Artificial ventilation	0.23 (0.14-0.41)	<.001	0.30 (0.09-0.95)	.06
	Chest compression	0.29 (0.17-0.49)	<.001	0.29 (0.1-0.86)	.04

**Table 3:** Odds ratios of performing procedures in pattern A (without family present) versus pattern B (with family present) by experience (N=454).

Vignette and procedure		Experienced (n=289)		Nonexperienced (n=165)	
		Odds ratio (95% CI)	*P* value	Odds ratio (95% CI)	*P* value
**Vignette 1**					
	Dialysis	0.43 (0.26-0.71)	<.001	0.80 (0.44-1.48)	.53
	Artificial ventilation	0.46 (0.29-0.74)	.001	0.63 (0.34-1.16)	.16
**Vignette 2**					
	Artificial ventilation	0.52 (0.30-0.90)	.02	0.73 (0.37-1.44)	.39
**Vignette 3**					
	Wound treatment	0.40 (0.21-0.76)	.004	0.78 (0.34-1.78)	.67
	Surgery	0.29 (0.16-0.53)	<.001	0.49 (0.22-1.08)	.08
	Blood transfusion	0.39 (0.24-0.63)	<.001	0.59 (0.32-1.10)	.12
	Vasopressor	0.43 (0.26-0.69)	<.001	0.62 (0.33-1.16)	.16
	Dialysis	0.30 (0.17-0.53)	<.001	0.56 (0.28-1.13)	.12
	Artificial ventilation	0.19 (0.10-0.35)	<.001	0.39 (0.18-0.87)	.02
	Chest compression	0.24 (0.13-0.43)	<.001	0.42 (0.19-0.93)	.03

**Table 4:** Odds ratios of performing procedures in pattern A (without family present) versus pattern B (with family present) by type of institution (N=454).

Vignette and procedure		Acute care hospital (n=264)		Non-acute care hospital (n=190)	
		Odds ratio (95% CI)	*P* value	Odds ratio (95% CI)	*P* value
**Vignette 1**					
	Dialysis	0.65 (0.40-1.06)	.11	0.43 (0.24-0.78)	.01
	Artificial ventilation	0.59 (0.36-0.97)	.05	0.41 (0.23-0.74)	.003
**Vignette 2**					
	Artificial ventilation	0.67 (0.38-1.17)	.21	0.49 (0.26-0.95)	.04
**Vignette 3**					
	Wound treatment	0.85 (0.44-1.63)	.74	0.25 (0.11-0.59)	.001
	Surgery	0.39 (0.21-0.71)	.003	0.31 (0.14-0.67)	.004
	Blood transfusion	0.53 (0.32-0.88)	.02	0.37 (0.21-0.68)	.002
	Vasopressor	0.65 (0.39-1.08)	.13	0.35 (0.19-0.63)	<.001
	Dialysis	0.45 (0.26-0.79)	.01	0.30 (0.15-0.60)	<.001
	Artificial ventilation	0.23 (0.12-0.46)	<.001	0.25 (0.12-0.52)	<.001
	Chest compression	0.33 (0.18-0.61)	<.001	0.25 (0.12-0.51)	<.001

**Table 5:** Factors associated with withholding of artificial ventilation.

Vignette and factors			Univariate analysis		Multivariate analysis	
			Odds ratio (95% CI)	*P* value	Odds ratio (95% CI)	*P* value
**Vignette** 1						
	**Sex**			.11		—^a^
		Female	1		—	
		Male	1.47 (0.91-2.37)		—	
	**Age**			.08		—
		<41	1		—	
		≥41	1.44 (0.98-2.12)		—	
	**Years since graduation**			.03		.03
		<17	1		1	
		≥17	1.55 (1.06-2.25)		1.54 (1.05-2.25)	
	**Institution**			.13		—
		Acute care hospital	1		—	
		Clinic	0.76 (0.48-1.2)		—	
		Chronic care hospital	1.66 (0.93-3.05)		—	
		Others	0.82 (0.39-1.7)		—	
	**Number of explanations**			.89		—
		0	1		—	
		1-6	1.09 (0.43-2.78)		—	
		7-12	1.41 (0.51-3.93)		—	
		≥13	1.12 (0.84-2.25)		—	
	**Department**			.30		—
		Internal medicine	1		—	
		Surgery	0.82 (0.41-1.61)		—	
		Orthopedics	2.07 (0.98-4.62)		—	
		Psychiatry	1.38 (0.65-3.03)		—	
		Others	1.03 (0.68-1.58)		—	
	**Pattern**			<.001		<.001
		A (without family)	1		1	
		B (with family)	0.51 (0.35-0.74)		0.51 (0.35-.75)	
**Vignette 2**						
	**Sex**			.29		—
		Female	1		—	
		Male	1.37 (0.81-2.29)		—	
	**Age**			.37		—
		<41	1		—	
		≥41	1.25 (0.81-1.92)		—	
	**Years since graduation**			.28		—
		<17	1		—	
		≥17	1.29 (0.85-1.97)		—	
	**Institution**			.009		—
		Acute care hospital	1		1	
		Clinic	0.67 (0.40-1.11)		0.66 (0.40-1.10)	.10
		Chronic care hospital	2.89 (1.27-7.78)		2.84 (1.25-7.67)	.02
		Others	0.64 (0.3-1.43)		0.57 (0.26-1.29)	.16
	**Number of explanations**			.11		—
		0	1		—	
		1-6	0.67 (0.22-1.83)		—	
		7-12	2.40 (0.64-9.38)		—	
		≥13	0.95 (0.34-2.36)		—	
	**Department**			.97		—
		**Internal medicine**	1		—	
		Surgery	0.89 (0.42-1.99)		—	
		Orthopedics	1.10 (0.48-2.75)		—	
		Psychiatry	0.83 (0.37-2.02)		—	
		Others	0.88 (0.54-1.43)		—	
	**Pattern**			.02		.01
		A (without family)	1		1	
		B (with family)	0.59 (0.39-0.90)		0.56 (0.36-0.87)	
**Vignette 3**						
	**Sex**			.85		—
		Female	1		—	
		Male	0.91 (0.49-1.60)		—	
	**Age**			.79		—
		<41	1		—	
		≥41	0.91 (0.57-1.45)		—	
	**Years since graduation**			.68		—
		<17	1		—	
		≥17	0.88 (0.56-1.39)		—	
	**Institution**			.42		—
		Acute care hospital	1		—	
		Clinic	0.74 (0.43-1.28)		—	
		Chronic care hospital	1.43 (0.69-3.28)		—	
		Others	0.79 (0.35-1.96)		—	
	**Number of explanations**			.21		—
		0	1		—	
		1-6	1.84 (0.67-4.93)		—	
		7-12	2.47 (0.8-7.86)		—	
		≥13	2.4 (0.97-5.65)		—	
	**Department**			.65		—
		Internal medicine	1		—	
		Surgery	2.01 (0.80-6.15)		—	
		Orthopedics	0.94 (0.41-2.37)		—	
		Psychiatry	1.00 (0.42-2.65)		—	
		Others	0.92 (0.56-1.53)		—	
	**Pattern**			<.001		<.001
		A (without family)	1		1	
		B (with family)	0.25 (0.15-0.40)		0.25 (0.15-0.4)	

^a^Not applicable.

## Discussion

Physicians withheld artificial ventilation for a patient without family through all three vignettes. Males, both experienced and those working at non-acute care hospitals, had a tendency to withhold medical procedures. Multivariate analysis showed the absence of family was the only significant factor associated with physicians’ decision making regarding withholding artificial ventilation.

Withholding of artificial ventilation for a patient without family may be because discontinuation of mechanical ventilation is not legally authorized in Japan, and physicians are concerned about unresponsive wakefulness syndrome. Further, physicians may apply their preference to a lonely, incapacitated patient. In Japan, only 5% of people have an advance directive, and 73% of people die in hospital [9,10]. The attending physician at the end of life is often different from the usual family doctor and has little chance to know the preference of the incapacitated patient without relatives. Fewer physicians would want to receive cardiopulmonary resuscitation (CPR) at the end of life compared with the general public. A survey by the Japanese government in 2018 reported that the percentage of people in the general public who did not want CPR in case of terminal cancer, severe heart disease, and advanced dementia was 69.2%, 67.4%, and 71.9%, respectively; however, this was 89.3%, 87.6%, and 90.1%, respectively, among physicians [10].

There is also a possibility that physicians provide unnecessary medical intervention to patients with family to satisfy their sentiment and avoid litigation. In vignette 3, 30% of physicians answered that they would prioritize the will of the family over the will of the patient and perform invasive procedures such as surgery or CPR. Under traditional Japanese culture, patients’ self-determination is sometimes in subordination to the will of the family; the Act on Organ Transplantation in Japan prohibits using organs of patients whose family members refused to offer, even when the patient themselves have a will to donate and have a donor card. As well, a previous study reported that among 934 patients with end-stage heart failure, only 4.7% of patients participated in end-of-life conversations with attending physicians and declared their preferences; surrogates made medical care decisions in 95.3% of cases [11].

Our study has some limitations. First, the patients were hypothetical; thus, this study does not demonstrate effects in daily clinical practice. Second, the response rates of online surveys tend to be lower than the response rates of face-to-face surveys [12-14]. Third, compared with the entire population of physicians in Japan, we assumed that fewer physicians in our study were working in a clinic, aged 20 to 29 years, or 60 years and older. However, this limitation would not considerably affect our main findings because approximately 74% of Japanese die in a hospital, and the last decisions regarding patient care are made in the hospital [15]. Further, physicians aged 20 to 29 years in training and 60 years or older in a supervising position do not usually work as an attending physician. Fourth, the majority of respondents were male, and this deviation can influence decision-making patterns [16]. Fifth, the view of death depends on the culture, and our results cannot be generalized to other countries directly. However, in the United States, it is estimated that there will be more than 2 million adults aged 70 years and older who have outlived all their friends and family members by 2030, so the implications of this survey do cross borders [4].

In conclusion, our findings suggest that elderly patients may sometimes be submitted to unwanted treatment due to the influence of family members or may have treatments withheld due to the absence of family. This highlights the potential importance of advance care planning in this era of an aging society with a declining birth rate.

## Appendix

Multimedia Appendix 1 Vignette A.

Multimedia Appendix 2 Vignette B.

## Abbreviations

OR: odds ratio
CPR: cardiopulmonary resuscitation
