# Supplementary material for: Physician Decision-Making Patterns and Family Presence: Cross-Sectional Online Survey Study in Japan
Source: Interact J Med Res. 2019 Sep 6;8(3):e12781. doi: 10.2196/12781 (PMC6764328; doi:10.2196/12781)
Supplement: Multimedia Appendix 1 [file ijmr_v8i3e12781_app1.pdf]

Vignette 1

Patient is a 65-years-old man with Child-Pugh C alcoholic liver cirrhosis and chronic kidney disease (serum creatinine was 4.5 mg/dl) who had often broke an appointment with doctor and did not take prescribed medicine. He has no spouse, is insulated from his relatives and lives alone. He was laying on the street and taken to a hospital by an ambulance. His Japan Coma Scale on admission was II-20, which is almost equal to the Glasgow Coma Scale score of 9 (E2V3M4). Body temparature was 38.8℃、blood pressure was 78/35 mmhg、heart rate was 130、saturation was 82% (Room air). He was diagnosed with pneumonia.

Could you please answer whether you would perform the described procedure by marking yes or no?

1) Oxygen
2) Blood culture
3) Lumbar puncture
4) Computed tomography
5) Antibiotics
6) Abdominocentesis
7) Blood transfusion
8) Central line
9) Vasopressor
10) Dialysis
11) Artificial ventilation
12) Chest compression

Vignette 2

Patient is a 78-years-old woman with severe dementia, chronic heart failure and osteoporosis, who lives in nursing home. Last year she suffered a fracture of the femur due to a fall and conservative treatment was done. Since them, her ADL (Activities of Daily Living) has weakened and she became a bedridden. Se has neither family nor relatives and was alone in the world. She was taken to a hospital by an ambulance and diagnosed with pneumonia. Her Japan Coma Scale on admission was II-20, whic is almost

equal to the Glasgow Coma Scale score of 9 (E2V3M4). Body temparature was 38.8℃、blood pressure was 78/35 mmhg、heart rate was 130、saturation was 82% (Room air).

Could you please answer whether you would perform the described procedure by marking yes or no?

1) Oxygen
2) Blood culture
3) Lumbar puncture
4) Computed tomography
5) Antibiotics
6) Blood transfusion
7) Central line
8) Vasopressor
9) Dialysis
10) Artificial ventilation
11) Chest compression

Vignette 3

Patient is a 70-years-old woman with arteriosclerosis obliterans, diabetes mellitus, hypertension, hyperlipidemia and thoracic aortic aneurysm repaired with endovascular stent-grafts who developed necrosis and ischemia of the lower limb with infection, which needed above-knee amputation to save her life. Patient wanted neither amputation nor cardiopulmonary resuscitation and understood the risk of death if she did not receive the amputation. Se has neither family nor relatives and was alone in the world.

Could you please answer whether you would perform the described procedure by marking yes or no?

1) Antibiotics
2) Wound treatment
3) Surgery
4) Blood transfusion
5) Vasopressor

6) Dialysis

7) Artificial ventilation

8) Chest compression
